# Qualitative Research with International Medical Graduates on the Impact of Cultural Differences on Psychotherapy Practice and Training

**DOI:** 10.1192/bjo.2026.11231

**Published:** 2026-06-30

**Authors:** Akshita Dandawate, Simon Graham, Jehan Elturky

**Affiliations:** Mersey Care NHS Trust, Liverpool, United Kingdom

## Abstract

**Aims:**

Previous studies have explored how international medical graduates (IMG) may face some barriers when it comes training in psychotherapy. This relates to cultural barriers when interacting with the patient or colleagues, or when working within a different system such as the National Health Service (NHS).

However few studies have explored their attitudes towards psychotherapy and how cultural factors may impact the way they think about therapy, training and the therapeutic alliance. Therefore, the aim of this study is to analyse transcripts from focus groups with IMGs to see what their thoughts are about psychotherapy and the training they received, and whether it is impacted by any cultural factors.

**Methods:**

Initially, all IMGs were invited to join focus groups via email and 8 volunteered to take part. The inclusion criteria were: trainees were currently at a placement in psychiatry in Mersey care and needed to have gained their medical degree outside of the UK. Two focus groups were held, using semi-structured questions. Themes were allowed to emerge but also prompted by pre-planned questions. The interviews were recorded, transcribed and analysed using thematic analysis by three separate readers. Different themes emerging were identified and coded.

**Results:**

Results showed themes that were pertinent to all trainees regardless of cultural background and these included: the importance of psychotherapy to practice and training, lacking confidence in its delivery and acknowledgement of the need for psychotherapy training and supervision.

However, there were some cultural impacts on their views of psychotherapy. It was highlighted that psychotherapy as practice is more widely accepted in the UK and that perhaps there is still stigma associated with mental health illnesses worldwide. There may be religious and social structures that can support people in other cultures which may impact how patients seek care from the medical profession and how professionals respond. There were positive and negative aspects from cultural differences identified that impacted upon the therapeutic alliance, the ways IMGs deliver therapy and what they were expecting from training. The existence of paternalistic practices internationally was raised by many, with debate as to the balance required with this and whether it can serve to be a positive influence at times.

**Conclusion:**

In conclusion, though cultural differences were identified that impacted IMG’s attitudes and delivery of therapy, they could be positive as well as negative. Solutions in the future, could perhaps be acknowledging and exploring these differences to strengthen delivery of therapy.

